# Impact of idiopathic pulmonary fibrosis on clinical outcomes of lung cancer patients

**DOI:** 10.1038/s41598-021-87747-1

**Published:** 2021-04-15

**Authors:** Ho Cheol Kim, Seonjeong Lee, Jin Woo Song

**Affiliations:** 1 Department of Pulmonary and Critical Care Medicine, Asan Medical Center, University of Ulsan College of Medicine, 88 Olympic-ro 43-gil, Songpa-gu, Seoul, 05505 Republic of Korea; 2 University of Ulsan College of Medicine, Seoul, Republic of Korea

**Keywords:** Non-small-cell lung cancer, Small-cell lung cancer, Risk factors, Outcomes research

## Abstract

The clinical characteristics of lung cancer in patients with idiopathic pulmonary fibrosis (IPF) differ from those of lung cancer in patients without IPF. Thus, we aimed to evaluate the impact of IPF on the clinical course of patients with lung cancer. Clinical data of IPF patients with lung cancer (n = 122) were compared with those of patients with lung cancer without IPF (n = 488) matched by age, sex, histopathology, stage, and date of diagnosis of lung cancer. The median follow-up period after diagnosis of lung cancer was 16 months. Among patients with IPF, the mean age was 68 years, 95.9% were male, 93.2% were ever-smokers, and squamous cell carcinoma was the most common cancer type (48.4%). The IPF group had poorer lung function and lower lobe predominance of lung cancer than the no-IPF group. The IPF group showed a poorer prognosis than the no-IPF group (5-year survival rate: 14.5% vs. 30.1%, respectively; *P* < 0.001), even after adjusting for lung function and regardless of the treatment method. Among patients with IPF, 16.8% experienced acute exacerbation within 1 month after treatment of lung cancer. The treatment outcome of patients with lung cancer and IPF was generally unfavorable, and acute exacerbation triggered by treatment frequently occurred.

## Introduction

Idiopathic pulmonary fibrosis (IPF) is characterized by progressive parenchymal fibrosis of unknown etiology and is associated with poor prognosis^1^. Patients with IPF have a median survival of 3 years, which is comparable to that of patients with cancer^2,3^. Lung cancer is a common complication of IPF^4,5^, with an incidence of approximately 22.9 per 10,000 person-years, which is approximately five times that seen in the general population^6^. Recently, Lee et al. also reported that patients with IPF (n = 25,241) have a higher lung cancer incidence (hazard ratio [HR], 5.89) than matched controls (n = 75,723) in Korea^7^. Additionally, Yoo et al. reported that in 938 Korean patients with IPF without lung cancer, the cumulative incidence of lung cancer was 31.1% after 10 years^8^.

Although several studies have suggested possible mechanisms for the association of IPF and lung cancer^9,10^, the optimal care for these patients has not been well investigated. The clinical characteristics of lung cancer in patients with IPF differ from those in patients without IPF. Patients with lung cancer and IPF are older and more frequently male smokers than those without IPF^11,12^. While the most common histopathologic subtype of lung cancer in the general population is adenocarcinoma^13^, squamous cell carcinoma (SqCC) is the most common type of cancer in patients with IPF^11,12,14^. Furthermore, patients with lung cancer and IPF tend to have poorer prognosis than patients with lung cancer without IPF, regardless of treatment modality^15–18^. However, previous studies have several limitations, including a small number of patients^19^, a lack of a control group^20^, and the use of specific subgroups, such as patients undergoing surgery or having a specific histopathology^14,21,22^. Therefore, we aimed to evaluate the impact of IPF on the clinical outcomes of patients with lung cancer using a large number of patients with IPF and matched controls.

## Materials and methods

### Study population

Among 893 patients with IPF diagnosed between January 2007 and December 2015 at the Asan Medical Center, Seoul, South Korea, 122 (13.7%) patients who developed lung cancer were included in this study. All patients with IPF met the diagnostic criteria of the American Thoracic Society (ATS)/European Respiratory Society (ERS)/Japanese Respiratory Society/Latin American Thoracic Association statement^2^. Patients with Lung cancer without IPF were randomly selected from the lung cancer registry to serve as controls; these patients were matched 1:4 by age at diagnosis of lung cancer, gender, histopathologic subtype, stage, and date of the lung cancer diagnosis. Patients were classified according to the histopathologic subtypes: adenocarcinoma, SqCC, small cell lung cancer (SCLC), and miscellaneous. Cancer stage for non-small cell lung cancer (NSCLC) was based on the 7th TNM staging system of the International Association for the Study of Lung Cancer^23^. Cancer stage for SCLC was based on the two-stage system originally introduced by the Veterans’ Affairs Lung Study Group^24^. The study was approved by the Institutional Review Board of Asan Medical Center (No.: 2015-0288), and informed consent was waived due to the retrospective nature of this study by the Institutional Review Board of Asan Medical Center. All methods were performed in accordance with the relevant guidelines and regulations.

### Clinical data

Clinical and survival data for all patients were obtained from medical records, telephone interviews, and/or the records of the National Health Insurance of Korea. Spirometry^25^, total lung capacity by plethysmography^26^, and diffusing capacity of the lung for carbon monoxide (DLco)^27^ were measured according to the ATS/ERS recommendation, and the results were expressed as a percentage of normal predicted values. The tumor location was determined based on chest computed tomography images and classified into upper/middle lobe or lower lobe predominance. Patients with tumor in both locations were considered as the lower lobe predominance group. Genetic mutations of lung cancer, including epidermal growth factor receptor (EGFR), KRAS, and anaplastic lymphoma kinase, were only analyzed in patients with adenocarcinoma. Resectable cases were defined as patients with stage I-IIIA NSCLC. Because surgical treatment of N2-positive stage IIIA tumors is still controversial^28,29^, these patients were excluded from the resectable case group. Acute exacerbation (AE) of IPF was defined using the criteria of Collard et al.^30^. AE triggered by treatment was defined as AE that occurred within 1 month after the last treatment.

### Statistical analysis

All values were presented as mean ± standard deviation for continuous variables and percentages for categorical variables. The chi-square test or Fisher’s exact test was used for categorical data, and the unpaired t-test was used for continuous data. Survival was assessed using Kaplan–Meier survival analysis and the log-rank test. Cox regression analysis was used to adjust forced vital capacity (FVC) in survival analysis. All *P*-values were two-tailed, with statistical significance set at *P* < 0.05. All statistical analyses were performed using SPSS 22.0 software (IBM Corporation, Armonk, NY, USA).

## Results

### Baseline characteristics

Among patients with IPF and lung cancer (n = 122), the mean age was 68.0 years, 95.9% were males, and 95.3% were ever-smokers. The median follow-up period after lung cancer diagnosis was 18.7 months. The baseline clinical characteristics of patients with NSCLC are shown in Table 1. Patients with IPF frequently experienced cough, dyspnea, and lower lung function than the no-IPF group. In addition, tumors were frequently located in the lower lobe in the IPF group compared to the no-IPF group. However, smoking history, and the proportion of gene mutations in patients with adenocarcinoma did not differ between the two groups. Baseline clinical characteristics of patients with SCLC are summarized in Table 2. As seen in the patients with NSCLC, patients with SCLC and IPF had lower pulmonary function, and tumors tended to be located more in the lower lobe.

**Table 1:** Comparison of the baseline characteristics of NSCLC between the IPF and no-IPF groups.

Characteristics	IPF	No-IPF	P-value
Number of patients	104	416	
Age, years	67.6 ± 6.7	68.2 ± 6.8	0.404
Male	100 (96.2)	400 (96.2)	> 0.999
Ever-smoker	85 (94.4)	339 (92.6)	0.544
BMI, kg/m^2^	23.5 ± 2.6	23.0 ± 3.2	0.165
Cough	65 (65.0)	189 (48.6)	0.003
Dyspnea	69 (69.0)	111 (29.1)	< 0.001
**PFT**			
FVC, % predicted	74.1 ± 15.1	81.4 ± 15.9	< 0.001
DLco, % predicted	54.5 ± 17.1	73.6 ± 24.2	< 0.001
TLC, % predicted	74.8 ± 11.3	91.1 ± 16.9	< 0.001
**Histologic subtypes**			> 0.999
Adenocarcinoma	39 (32.0)	156 (32.0)	
SqCC	59 (48.4)	236 (48.4)	
Miscellaneous^a^	6 (4.9)	24 (4.9)	
**Clinical stage**			> 0.999
Stage I	48 (46.2)	192 (46.2)	
Stage II	12 (11.5)	48 (11.5)	
Stage III	19 (18.3)	76 (18.3)	
Stage IV	25 (24.0)	100 (24.0)	
**Location (lobe)**			0.028
Upper or middle	45 (43.7)	226 (55.8)	
Lower	58 (56.3)	179 (44.2)	
**Gene mutation**^b^			
EGFR (n = 28/77)	9 (32.1)	26 (33.8)	0.876
KRAS (n = 18/53)	1 (5.6)	10 (18.9)	0.177
ALK (n = 19/61)	0 (0)	2 (3.3)	0.424

Data are presented as mean ± SD or number (%), unless otherwise indicated.

*NSCLC* non-small cell lung cancer, *IPF* idiopathic pulmonary fibrosis, *BMI* body mass index, *PFT* pulmonary function test, *FVC* forced vital capacity, *DLco* diffusing capacity for carbon monoxide, *TLC* total lung capacity, *SqCC* squamous cell carcinoma, *EGFR* epidermal growth factor receptor, *ALK* anaplastic lymphoma kinase.

^a^Miscellaneous: IPF (3 adenosquamous carcinomas, 1 large cell carcinoma, 2 sarcomatoid carcinomas), No-IPF (11 adenosquamous carcinomas, 4 large cell carcinomas, 3 sarcoid carcinomas, 1 sarcomatoid carcinoma, 2 malignant spindle cell carcinomas, 1 adenoid carcinoid tumor, 1 atypical carcinoid tumor, 1 pleomorphic carcinoma).

^b^Gene mutation: gene mutation profile was analyzed only for adenocarcinoma.

**Table 2:** Comparison of the baseline characteristics of SCLC between patients in the IPF and no-IPF groups.

Characteristics	IPF	No-IPF	P-value
Number of patients	18	72	
Age, years	70.4 ± 7.6	70.0 ± 6.9	0.828
Male	17 (94.4)	68 (94.4)	> 0.999
Ever-smoker	16 (100.0)	57 (95.0)	0.487
BMI, kg/m^2^	23.9 ± 2.7	23.3 ± 3.4	0.543
Cough	16 (88.9)	39 (57.4)	0.013
Dyspnea	13 (76.5)	30 (45.5)	0.022
**PFT**			
FVC, % predicted	65.3 ± 13.4	73.3 ± 13.6	0.055
DLco, % predicted	46.1 ± 13.4	65.7 ± 18.1	< 0.001
TLC, % predicted	64.3 ± 13.5	82.5 ± 17.0	0.006
**Stage**			> 0.999
Limited	3 (16.7)	12 (16.7)	
Extensive	15 (83.3)	60 (83.3)	
**Location of cancer (lobe)**			0.111
Upper or middle	6 (33.3)	36 (54.5)	
Lower	12 (66.7)	30 (45.5)	

Data are presented as mean ± SD or number (%), unless otherwise indicated.

*SCLC* small cell lung cancer, *IPF* idiopathic pulmonary fibrosis, *BMI* body mass index, *PFT* pulmonary function test, *FVC* forced vital capacity, *DLco* diffusing capacity for carbon monoxide, *TLC* total lung capacity.

### Treatment

Treatments in patients with NSCLC are summarized in Table 3. The proportions of patients who underwent surgery, chemotherapy, and radiation therapy were similar between the IPF and no-IPF groups. Among patients who underwent surgical treatment, the proportion that underwent sublobar resection (wedge resection or segmentectomy) was higher in the IPF group than in the no-IPF group (41.8% vs. 11.2%, *P* < 0.001). Among patients who underwent radiation therapy, the IPF group underwent more localized treatment, such as stereotactic radiosurgery (SRS) or stereotactic body radiotherapy (SBRT), than the no-IPF group (52.2% vs. 24.6%, *P* = 0.015). Treatments in patients with resectable and non-resectable NSCLC are summarized in Supplementary Tables S1 and S2, respectively. While the IPF group frequently underwent sublobar resection and stereotactic radiosurgery compared to the no-IPF group among patients with resectable NSCLC, there were no differences in treatment between the IPF and no-IPF groups among patients with unresectable NSCLC. Among patients with SCLC, there were no differences in treatments between the IPF and no-IPF groups (Table 4).

**Table 3:** Comparison of treatment of NSCLC between the IPF and no-IPF groups.

Characteristics	IPF	No-IPF	P-value
Number of patients	104	416	
Resectable case	66 (63.5)	267 (64.2)	0.891
Surgery	55 (52.9)	197 (47.4)	0.313
**Types of surgery**			< 0.001
Lobar resection^a^	32 (58.2)	175 (88.8)	
Sublobar resection^b^	23 (41.8)	22 (11.2)	
Chemotherapy	33 (31.7)	134 (32.2)	0.925
Target therapy^c^ (n = 39/156)	4 (10.1)	32 (20.5)	0.140
**Target therapy regimen**			0.319
Gefitinib	3 (75.0)	19 (59.4)	
Erlotinib	0	9 (28.1)	
Gefitinib + Erlotinib	0	2 (6.3)	
Afatinib	1 (25.0)	1 (3.1)	
Crizotinib	0	1 (3.1)	
Radiation therapy	23 (22.1)	65 (15.6)	0.114
**Types of RT**			0.015
SRS or SBRT	12 (52.2)	16 (24.6)	
Conventional RT	11 (47.8)	49 (75.4)	

Data are presented as number (%), unless otherwise indicated.

*NSCLC* non-small cell lung cancer, *IPF* idiopathic pulmonary fibrosis, *RT* radiation therapy, *SRS* stereotactic radiosurgery, *SBRT* stereotactic body radiotherapy.

^a^Lobar resection: lobectomy or more extensive resection (bilobectomy or pneumonectomy).

^b^Sublobar resection: segmentectomy or wedge resection.

^c^Information from only adenocarcinoma patients.

**Table 4:** Comparison of treatment of SCLC between the IPF and no-IPF groups.

Characteristics	IPF	No-IPF	P-value
Number of patients	18	72	
Surgery	3 (16.7)	9 (12.5)	0.700
**Types of surgery**			0.236
Lobar resection^a^	1 (66.7)	7 (77.8)	
Sublobar resection^b^	2 (33.3)	2 (22.2)	
Chemotherapy	13 (72.2)	60 (83.3)	0.318
Radiation therapy	5 (27.8)	28 (38.9)	0.832

Data are presented as number (%), unless otherwise indicated.

*SCLC* small cell lung cancer, *IPF* idiopathic pulmonary fibrosis.

^a^Lobar resection: lobectomy or more extensive resection (bilobectomy or pneumonectomy).

^b^Sublobar resection: segmentectomy or wedge resection.

### Survival according to histopathology or stage

**Figure 1:**
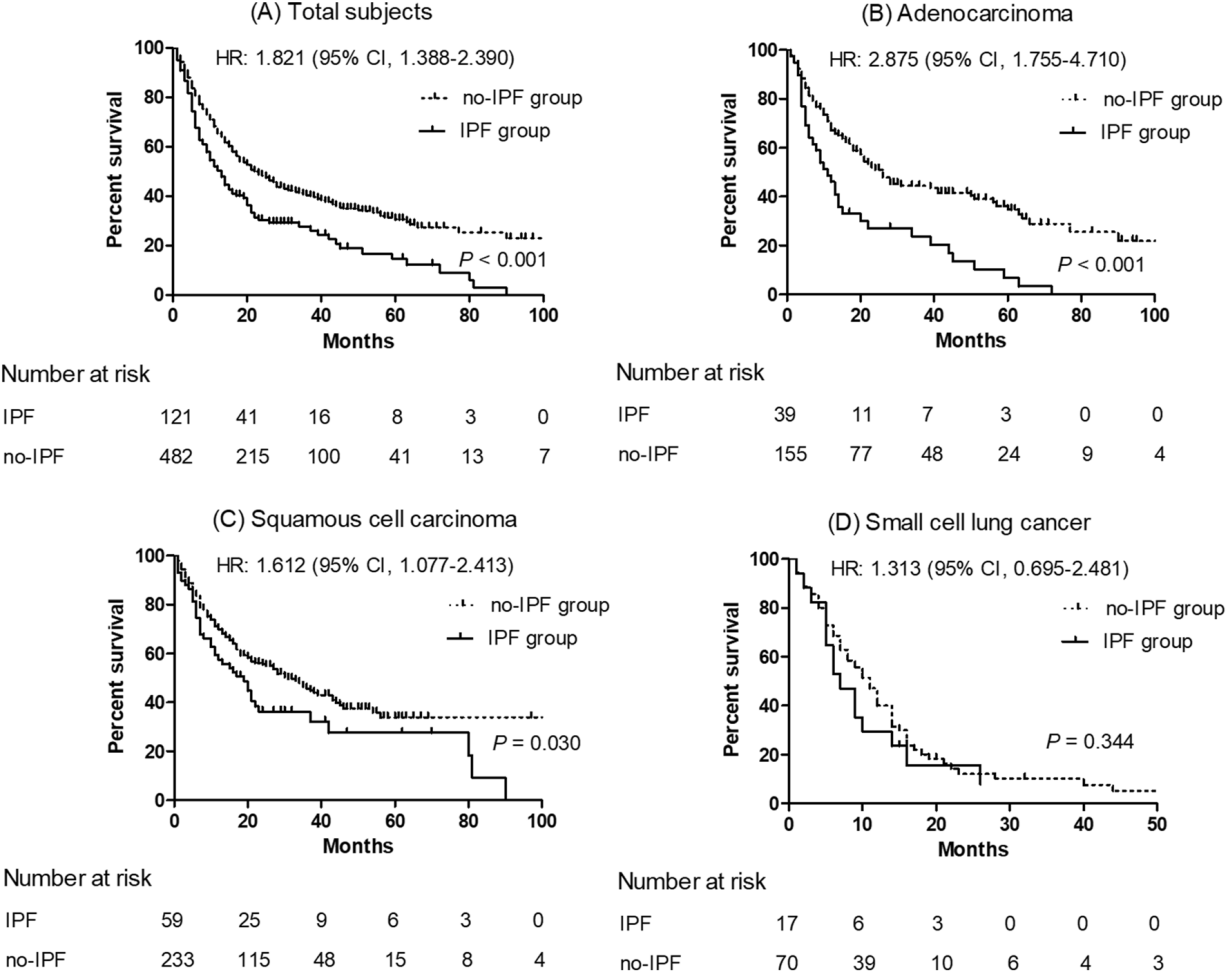
Comparison of survival curves between the IPF and no-IPF groups among patients with lung cancer. (**A**) Total lung cancer, (**B**) adenocarcinoma, (**C**) squamous cell carcinoma, and (**D**) small cell lung cancer.

Among patients with lung cancer, the IPF group had a poorer prognosis than the no-IPF group (5-year survival rate: 14.5% vs. 30.1%, *P* < 0.001) (Fig. 1). When classified according to histopathologic types of lung cancer, there was no difference in survival between the IPF and no-IPF groups in patients with SCLC; however, the IPF group was associated with poorer prognosis compared to the no-IPF group among patients with adenocarcinoma (median survival: 11 vs. 26 months, *P* < 0.001) or SqCC (median survival: 19 vs. 30 months, *P* = 0.003) (Fig. 1). When adjusted for FVC in the Cox regression analysis, IPF was independently associated with mortality in patients with lung cancer (HR 1.552; 95% CI 1.195–2.016; *P* = 0.001) or those with adenocarcinoma (HR 2.165; 95% CI 1.375–3.408; *P* = 0.001).

**Figure 2:**
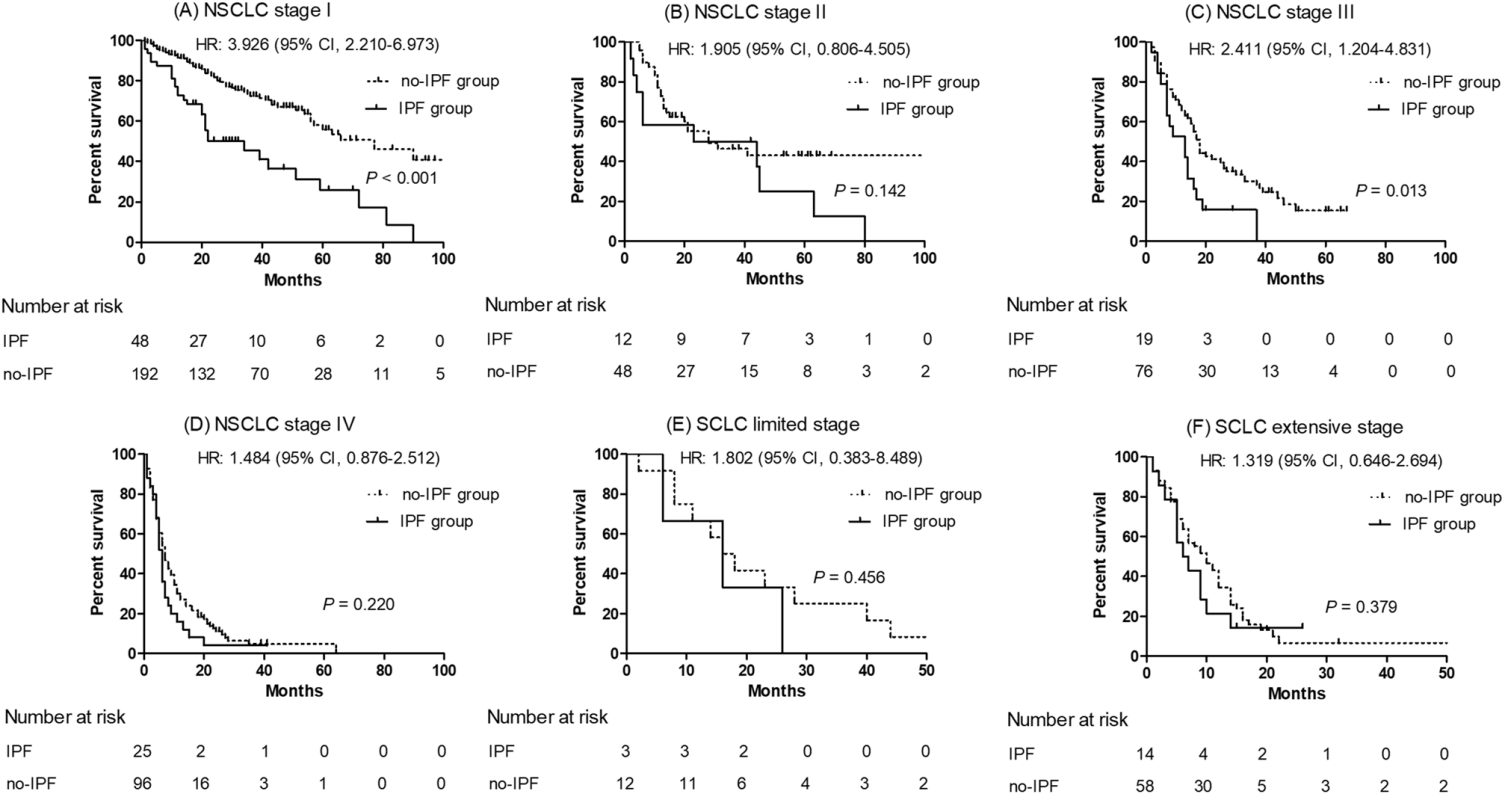
Comparison of survival curves between the IPF and no-IPF groups among patients with lung cancer. (**A**) NSCLC stage I, (**B**) NSCLC stage II, (**C**) NSCLC stage III, (**D**) NSCLC stage IV, (**E**) SCLC limited stage, and (**F**) SCLC extensive stage. *NSCLC* non-small cell lung cancer, *SCLC* small cell lung cancer.

Comparison of survival curves between patients with lung cancer and IPF and those without according to the clinical stage is shown in Fig. 2. The median survival of patients with lung cancer and IPF was shorter than that of the no-IPF group in stage I (34 vs. 77 months, *P* < 0.001) and III of NSCLC (13 months vs. 18 months, *P* = 0.013); these results also did not change after adjusting for FVC. However, the median survival between the IPF and no-IPF groups in stage II (23 months vs. 28 months, *P* = 0.142) and stage IV of NSCLC (6 months vs. 7 months, *P* = 0.220) was similar. Among patients with SCLC, the median survival of the IPF and no-IPF groups was similar in both limited (16 months vs. 16 months, *P* = 0.456) and extensive stages of SCLC (6 months vs. 9 months, *P* = 0.379).

### Survival according to treatment

Among patients with resectable NSCLC who underwent surgery, the IPF group had a shorter median survival period than the no-IPF group (42 months vs. 90 months, *P* < 0.001) (Supplementary Fig. S1A). The results did not change after adjusting for FVC in the Cox regression analysis (HR 2.911; 95% CI 1.812–4.675; *P* = 0.001). When classified according to the surgical method, the IPF group still had a poorer prognosis than the no-IPF group among patients who underwent sublobar resection (median survival: 22 months vs. not reached, *P* = 0.011) (Supplementary Fig. S1B) or lobar resection (median survival: 45 months vs. 90 months, *P* < 0.001) (Supplementary Fig. S1C).

Among patients with non-resectable NSCLC who underwent chemotherapy, the IPF group had shorter median survival than the no-IPF group (7 months vs. 11 months, *P* = 0.064) (Supplementary Fig. S2A). In the Cox analysis adjusted for FVC, the IPF group also had a poorer prognosis than the no-IPF group (HR 2.007; 95% CI 1.131–3.561; *P* = 0.017). Among patients with non-resectable NSCLC who underwent radiation therapy, the IPF group had a poorer prognosis than the no-IPF group (5 months vs. 18 months, *P* < 0.001; Supplementary Fig. S2B).

### Acute exacerbation triggered by treatment

Among 122 patients with lung cancer and IPF, 101 patients (82.8%) underwent anti-cancer treatment such as surgery, chemotherapy, and radiation therapy. Of these patients, 17 (16.8%) experienced AE within a month from the last treatment; six experienced AE after surgery (10.3% of patients with IPF who underwent surgery), six experienced AE after chemotherapy (13.0% of patients with IPF who underwent chemotherapy), and five experienced AE after radiation therapy (17.9% of patients with IPF who underwent radiation therapy). Among patients who underwent anti-cancer therapy, those who experienced AE had worse prognosis after cancer diagnosis (median survival period: 5 months vs. 16 months, *P* = 0.002) than those who did not experience AE (Supplementary Fig. S3).

## Discussion

In this study, among patients with lung cancer, the IPF group had a poorer prognosis than the no-IPF group, even after adjusting for IPF- or lung cancer-related prognostic variables including age, sex, clinical stage, and lung function. Treatment of lung cancer triggered AE in 16.8% of patients with IPF, leading to poor clinical outcome.

Lung cancer is an acknowledged comorbidity associated with IPF^18,19,31^, involving several possible mechanisms such as genetics, epigenetics, and cell signaling pathways^32^. SqCC remained the most frequent tumor subtype in patients with IPF, although adenocarcinoma is the most common tumor subtype in the general population^33,34^. Tzouvelekis et al., in 102 patients with IPF and lung cancer from a multicenter in Greece, reported that SqCC (34.3%) was the most common histologic subtype^35^. However, in a recent international survey performed by the ERS, participating physicians responded that adenocarcinoma (58.6%) was the most common histological type of lung cancer in patients with IPF, followed by SqCC (26.6%)^36^. In addition, the high proportion of male patients and lower lobe predominance are consistent with the results of previous studies^14,16,37^. Interestingly, the proportion of adenocarcinoma with the EGFR mutation was similar in the IPF and no-IPF groups in our study, which is in contrast to the results of previous studies that showed that patients with IPF had a lower EGFR mutation rate than no-IPF patients^38,39^. Masai et al. reported that patients with usual interstitial pneumonia (UIP)-adenocarcinoma (n = 44) had a lower EGFR mutation rate (2.3% vs. 45.6%, *P* < 0.01) than patients with non-UIP-adenocarcinoma (n = 2265)^38^. Additionally, Kanaji et al., in 218 patients with NSCLC, reported that while patients with IPF (n = 34) had no EGFR mutation, 32% of patients with non-interstitial lung disease (ILD) (n = 165) had EGFR mutation^39^. Further studies will be required to confirm this finding and determine the efficacy and safety of molecular targeted therapy on EGFR mutant adenocarcinoma in patients with IPF.

In this study, patients with lung cancer and IPF had poorer outcomes than those without IPF, and this result is consistent with those of previous reports^14,19,37^. Although the exact mechanisms that underlie these findings are still unclear, they may involve risk factors of lung cancer development such as old age, male gender, and smoking^5,19^. Several hypotheses about the common pathways of IPF and cancer have been presented, including genetic abnormality and altered intracellular signaling^32,40,41^. Moreover, Yoo et al., showed that rapid decline of FVC is associated with lung cancer development, which is also associated with poor outcomes in patients with IPF^8^. In our study, IPF was confirmed to be a poor prognostic factor even after controlling for age, gender, histopathologic subtype, stage, and date of diagnosis. Furthermore, these results did not change after adjusting for lung function irrespective of treatment method. In our study, there were no differences in survival between the IPF and no-IPF groups among patients with SCLC or advanced (stage IV) NSCLC. A recent international survey reported that palliative care (69%) was the most frequent management in both advanced IPF and lung cancer (TNM stage IV) cases^36^. However, Koyama et al., in 122 patients with SCLC, reported that the IPF group (n = 20, median survival 244 days) had shorter survival than the IIP (idiopathic interstitial pneumonia) other than IPF group (n = 27, median survival 386 days) or non-IIP group (n = 73, median survival 592 days, *P* = 0.001)^42^. This may be due to the higher proportion of patients with extensive-stage, which is associated with aggressive clinical course, in our cohort (83.3%) compared to previous studies (55%). Based on these findings, tailored management of patients with lung cancer and IPF is required to improve the poor prognosis of patients with IPF-lung cancer.

During the management of patients with IPF and lung cancer, the impact of treatment-related adverse events, such as postoperative pulmonary morbidity^15–17,43–45^, AE^21^, and radiation pneumonitis, should be considered^46^. Indeed, 16.8% of patients who underwent anti-cancer treatment experienced AE in our study. Previous studies showed that patients with lung cancer and IPF might experience postoperative AE and have a higher pulmonary morbidity rate after surgery than those without IPF^44,47–49^. Watanabe et al., in 870 patients with lung cancer, reported that 7.1% of patients in the IPF group (4/56) experienced postoperative AE, and postoperative acute respiratory distress syndrome was more common in the IPF group (7.1% vs. 0.9%, *P* < 0.004) than in the no-IPF group^44^. Saito et al., in 350 stage IA patients with NSCLC, showed that postoperative AE occurred in 10.7% of patients with IPF (n = 28)^48^. Aside from surgical treatment, Kenmotsu et al., in 189 patients with lung cancer with ILD who underwent chemotherapy, reported that approximately 30% of patients with UIP pattern (n = 21) experienced AE after treatment^49^. These findings suggest that careful monitoring of patients with lung cancer is needed even after treatment.

There were some limitations to this study. First, the retrospective study design may have resulted in selection bias. For example, some important clinical information (such as the cause of death) was available for only a few study participants. However, we included many patients with few missing data. Second, this study was performed at a single center in Korea. Therefore, it may not be possible to generalize our results to other settings. However, the baseline clinical characteristics of patients were similar to those in previous studies^19,39^. Finally, although the number of patients in this study was relatively large compared to previous studies, it is still too small to perform significant comparisons among specific conditions. However, we believe that the present case–control study may help to improve our understanding of the clinical course of lung cancer in patients with IPF and may serve as a reference for future studies.

In conclusion, our results showed that IPF affects clinical outcomes of patients with lung cancer irrespective of lung function or treatment methods, and AE occurred in a fifth of patients with IPF after lung cancer treatment. These findings suggest that treatment strategies tailored to patients with IPF and the prevention of AE are important for improving their prognosis.

## Supplementary Information

Supplementary Figure 1.

Supplementary Figure 2.

Supplementary Figure 3.

Supplementary Tables.

## Data Availability

The datasets generated during and/or analyzed during the current study are available from the corresponding author on reasonable request.

## Abbreviations

IPF: Idiopathic pulmonary fibrosis
SqCC: Squamous cell carcinoma
SCLC: Small cell lung cancer
NSCLC: Non-small cell lung cancer
FVC: Forced vital capacity
DLco: Diffusing capacity for carbon monoxide
TLC: Total lung capacity
PFT: Pulmonary function test
BMI: Body mass index
EGFR: Epidermal growth factor receptor
ALK: Anaplastic lymphoma kinase
