# Supplementary figures and images for: Impact of idiopathic pulmonary fibrosis on clinical outcomes of lung cancer patients
Source: Sci Rep. 2021 Apr 15;11:8312. doi: 10.1038/s41598-021-87747-1 (PMC8050293; doi:10.1038/s41598-021-87747-1)

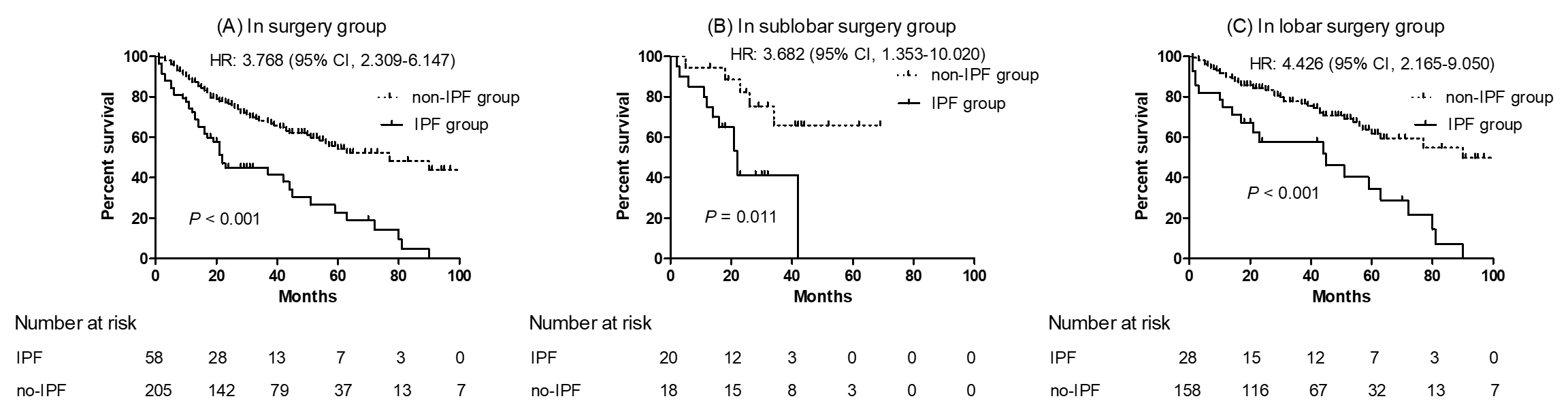

Supplement: Supplementary file 1 — Supplementary Figure 1. [file 41598_2021_87747_MOESM1_ESM.tiff]

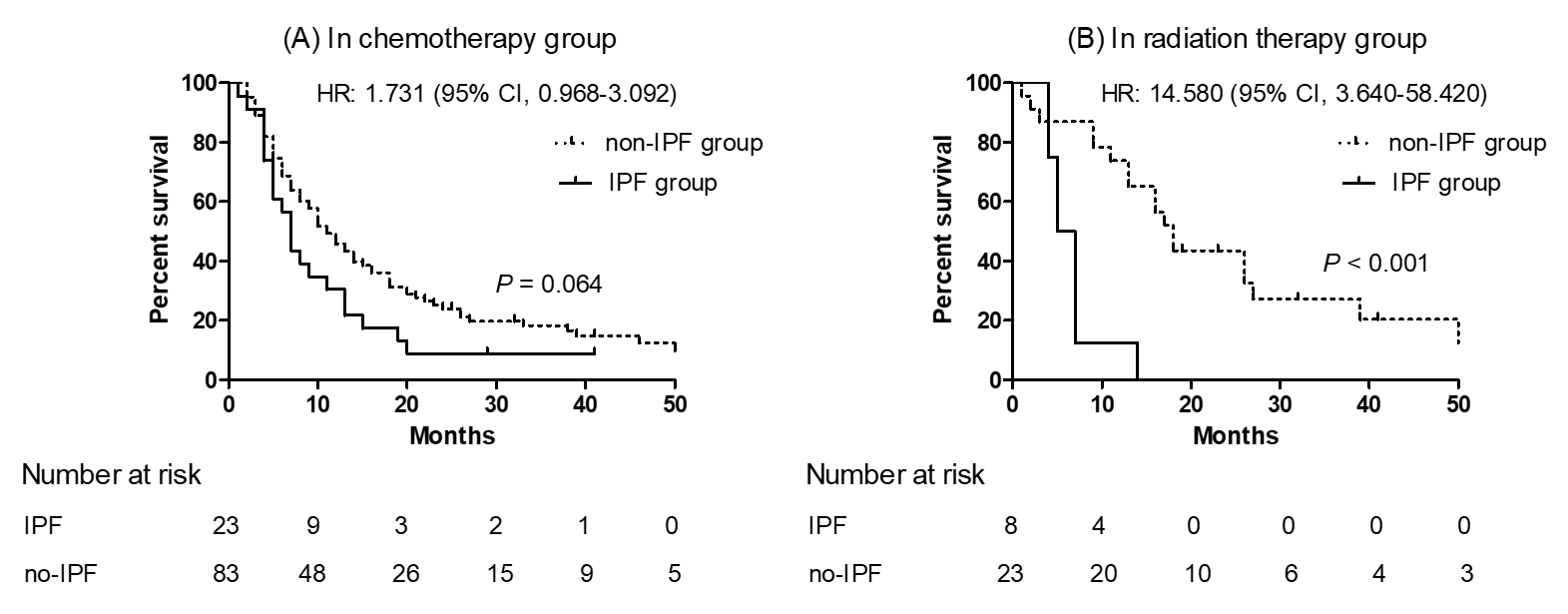

Supplement: Supplementary file 2 — Supplementary Figure 2. [file 41598_2021_87747_MOESM2_ESM.tiff]

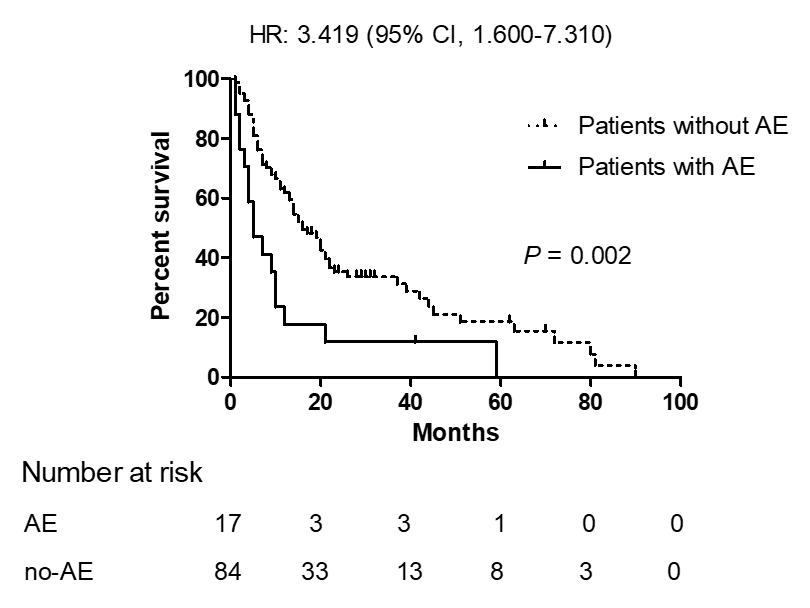

Supplement: Supplementary file 3 — Supplementary Figure 3. [file 41598_2021_87747_MOESM3_ESM.tiff]
