# Supplementary material for: Impact of idiopathic pulmonary fibrosis on clinical outcomes of lung cancer patients
Source: Sci Rep. 2021 Apr 15;11:8312. doi: 10.1038/s41598-021-87747-1 (PMC8050293; doi:10.1038/s41598-021-87747-1)
Supplement: Supplementary file 4 — Supplementary Tables. [file 41598_2021_87747_MOESM4_ESM.docx]

**Impact of Idiopathic Pulmonary Fibrosis on Clinical Outcomes of Lung Cancer Patients**

Ho Cheol Kim^1^, MD, Seonjeong Lee^2^, MD, Jin Woo Song, MD, PhD^1^

**Affiliations:**

^1^Department of Pulmonary and Critical Care Medicine, Asan Medical Center, University of Ulsan College of Medicine, Seoul, Republic of Korea

^2^University of Ulsan College of Medicine, Seoul, Republic of Korea

**Table S1. Comparison of treatment of resectable NSCLC between the IPF and no-IPF groups**

| Characteristics | IPF | no-IPF | p-value |
| --- | --- | --- | --- |
| Number of patients | 66 | 267 |  |
| Surgery | 48 (72.7) | 176 (65.9) | 0.291 |
| Surgical procedure |  |  | < 0.001 |
| Lobar resection* | 28 (58.3) | 158 (89.8) |  |
| Sublobar resection^†^ | 20 (41.7) | 16 (10.2) |  |
| Chemotherapy | 10 (15.2) | 51 (19.1) | 0.458 |
| Target therapy^¶^(n=6/19) | 0 (0) | 4 (21.1) | 0.540 |
| Radiation therapy | 15 (22.7) | 42 (15.7) | 0.177 |
| Types of RT |  |  | 0.001 |
| SRS or SBRT | 12 (80.0) | 13 (31.0) |  |
| Conventional RT | 3 (20.0) | 29 (69.0) |  |

Data are presented as number (%)unless otherwise indicated.

NSCLC: non-small cell lung cancer, IPF: idiopathic pulmonary fibrosis, RT: radiation therapy, SRS: stereotactic radiosurgery, SBRT: stereotactic body radiotherapy

* Lobar resection: Lobectomy or more extensive resection (bilobectomy or pneumonectomy).

†Sublobar resection: Segmentectomy or Wedge resection.

¶ Among adenocarcinoma patients with EGFR mutation or ALK mutation (n=25).

**Table S2. Comparison of treatment of unresectable NSCLC between the IPF and no-IPF groups**

| Characteristics | IPF | no-IPF | p-value |
| --- | --- | --- | --- |
| Number of patients | 38 | 149 |  |
| Chemotherapy | 23 (60.5) | 83 (55.7) | 0.592 |
| Target therapy* | 3/3 (100.0) | 5/8 (62.5) | 0.491 |
| Kind of target therapy |  |  | 0.449 |
| Gefitinib | 3 (100.0) | 3 (60.0) |  |
| Erlotinib | 0 | 1 (20.0) |  |
| Crizotinib | 0 | 1 (20.0) |  |
| Radiation therapy | 8 (21.1) | 23 (15.4) | 0.406 |

Data are presented as number (%), unless otherwise indicated.

NSCLC: non-small cell lung cancer, IPF: idiopathic pulmonary fibrosis

* Among adenocarcinoma patients with EGFR mutation or ALK mutation (n=11).

**Figure S1.** Comparison of survival curves between the IPF and no-IPF groups among patients with resectable NSCLC.

(A) Total surgery, (B) Sublobar surgery, and (C) Lobar surgery.

IPF: idiopathic pulmonary fibrosis, NSCLC: non-small cell lung cancer

**Figure S2.** Comparison of survival curves between the IPF and no-IPF groups among patients with unresectable NSCLC.

(A) Chemotherapy and (B) Radiation therapy.

IPF: idiopathic pulmonary fibrosis, NSCLC: non-small cell lung cancer

**Figure S3.** Comparison of survival curves according to development of AE in patients with lung cancer with IPF.

AE: acute exacerbation, IPF: idiopathic pulmonary fibrosis
